# A Novel Integrated and Labile eHealth System for Monitoring Dog Rabies Vaccination Campaigns

**DOI:** 10.3390/vaccines7030108

**Published:** 2019-09-09

**Authors:** Andre Coetzer, Terence P. Scott, Khadija Noor, Lambert F. Gwenhure, Louis H. Nel

**Affiliations:** 1 Department of Biochemistry, Genetics and Microbiology, Faculty of Natural and Agricultural Sciences, University of Pretoria, Pretoria 0002, South Africa; 2 Global Alliance for Rabies Control SA NPC, Pretoria 0181, South Africa; 3 Department of Livestock Development, Zanzibar Central Veterinary Laboratory, Maruhubi 71101, Tanzania; 4 Department of Livestock & Veterinary Services, Central Veterinary Research and Diagnostic Laboratory, Harare 04-263, Zimbabwe; 5 Global Alliance for Rabies Control, Manhattan, KS 66502, USA

**Keywords:** rabies, strategic dog vaccination, GARC Data Logger, Rabies Epidemiological Bulletin, Rabies Vaccination Tracker

## Abstract

The elimination of canine rabies through the implementation of high coverage mass dog vaccination campaigns is a complex task, particularly in the resource-limited countries of the rabies endemic world. Here we demonstrated the feasibility of applying targeted rabies vaccination campaigns to deliver more impactful intervention campaigns in resource-limited settings using evidence and lessons learnt from other diseases. With the use of strategic rabies intervention programs, we demonstrate the noteworthy reduction of rabies cases in two very different African settings. The strategic intervention was most significantly aided by the use of a custom-developed vaccination tracking device (the Global Alliance for Rabies Control (GARC) Data Logger) and an integrated rabies surveillance system (the Rabies Epidemiological Bulletin). Our first case study, an island-wide strategic dog vaccination on Tanzania’s Unguja island, reduced the incidence of rabies by 71% in the first 16 months of implementation. In the second case study, a similar approach was applied in the metropolitan capital city of Zimbabwe and the incidence of rabies declined by 13% during the first 13 months of implementation. The methodologies and results presented here suggest that, in resource-limited settings, an optimal approach towards the elimination of dog rabies would revolve around strategic interventions, subject to the use of appropriate planning, surveillance, and vaccination tools.

## 1. Introduction

Rabies is one of the oldest known zoonotic diseases [1]. Despite the fact that the first vaccine for *Rabies lyssavirus* (RABV)—the type species for the *Lyssavirus* genus—was developed as far back as the 1880s [2], rabies still has a near global distribution, with dog-mediated rabies causing approximately 59,000 human deaths every year [3,4]. The control and elimination of dog-mediated human rabies through the vaccination of dogs has been demonstrated in countries and regions with different social and political conditions across the world [3,5,6,7]. In these successful interventions, it has been shown that the vaccination of 70% of the total dog population is sufficient to interrupt disease transmission to the extent that elimination can be achieved [3]. While reaching this level of vaccination coverage is in principle a straightforward task, it is also time-consuming, resource-intensive, and financially cumbersome—thus relying on continuous governmental support and buy-in over the course of multiple years [8]. As an example, most Latin American and Caribbean (LAC) countries have been implementing annual mass dog vaccination (MDV) campaigns since the 1980s [5,9,10]. Through the continued commitment of the LAC governments, these long-term campaigns have managed to reduce the number of rabies cases in humans and dogs by 95% over the past 35 years—with dog rabies cases still sporadically detected in some of the countries [10,11].

Given the long-term commitment required, can resource-limited countries in Africa emulate the success of those countries that have to-date eliminated dog-mediated rabies? An array of studies suggest that this would be feasible, subject to national government prioritisation of the large and continued investment towards (i) the implementation of national MDV campaigns and (ii) all of the resources associated with the multi-year operational costs that are required to ensure programmatic success [8,12,13,14]. That being said, do the countries of rabies-endemic Africa have the means to implement sustainable intervention strategies of the scale, intensity, and duration that is required to reach at least 70% of the total dog population? To date, rabies control on the African continent continues to experience limited capital investment, with the estimated gap in funding required to ensure adequate vaccination coverage of the total dog population within the range of 79 million USD [8,15]. Capital investment, however, is not something that can be drastically improved in a short period of time, and alternative approaches towards implementing successful dog vaccination campaigns should thus be considered. These approaches should ideally rely on the optimal utilization of the existing resources in such a way that maximum effect may be achieved.

When considering strategies that have been implemented successfully for other diseases, one key element was paramount in all the cases that we have investigated. This commonality was simply a strategic intervention approach that relied on prioritized targeting of the at-risk population as opposed to the total population. In such a strategy, some surveillance data and knowledge of the disease epidemiology is critical to focus primarily on the areas where the disease is prevalent before progressing to populations that were considered at a lower risk. The role of such a targeted intervention approach in the eradication of rinderpest and the enhanced control of foot and mouth disease (FMD) and malaria is briefly discussed below to highlight the effectiveness of the strategic approach and use this information to make a clear case for its use in rabies elimination.

### 1.1. Rinderpest

Rinderpest is the second disease to be eradicated [16]. Since the 1960s, rinderpest eradication programs relied on annual mass vaccination programs aimed at covering the entire national population. This approach proved successful in most cases but was also found to be ineffective in many countries (e.g., Ethiopia, Uganda, and Sudan) as a result of pastoral behaviour and limited governmental resources. The challenge in theses settings was overcome by abandoning the blanket approach and shifting focus to those communities known to be infected. This strategic approach helped those lagging countries eradicate the disease within a few short years [16,17]. Importantly, the strategy was fully dependent on adequate surveillance and community involvement towards accurate identification of infected areas, without which targeted disease intervention would not have been implemented in a timely fashion. The successes from rinderpest have demonstrated that targeted vaccination based on good surveillance is, in fact, more feasible in resource-limited settings than mass vaccination, as, without intense international support, mass vaccination would be too costly and too great a burden on the current health system in these countries.

### 1.2. Foot and Mouth Disease (FMD)

Similar to both rinderpest and the current rabies situation, FMD was eliminated from most European countries between the 1950s and 1990 through large-scale interventions and mass vaccination campaigns [18]. These approaches, however, proved to be less effective in resource-limited countries due to their requirements for significant coordinative and financial resources [18]. As a result, a progressive, targeted, risk-based approach was developed for countries with limited resources [18,19]. This risk-based approach was found to be the most effective approach for controlling outbreaks and interrupting FMD transmission—subsequently becoming a key component to the national strategy for many endemic countries [20]. Thus, as rabies faces similar challenges, a strategic intervention approach seems logical for achieving success in resource-limited countries.

### 1.3. Malaria

For malaria, vector control remains the recommended prevention method in endemic countries. Vector control should ideally target all mosquito populations in an endemic country but this is logistically infeasible. As such, the core concept of the “Framework for Malaria Elimination” is that intervention strategies should be limited to targeted campaigns that respond to areas where malaria cases are reported, with intervention being prioritised based on the reported incidence rates [21,22]. This targeted approach has proven to be extremely successful, enabling 17 countries and territories to completely eliminate malaria since the year 2000 [22] and facilitating a 37% reduction in the global incidence [22]. Once again, successful malaria control programs illustrated that a disease that specifically targets poor and rural communities can be drastically reduced and eliminated in low- and middle-income states through strictly targeted interventions based on reliable and timely data, with comparable outcomes to the more costly mass vaccination/intervention campaigns.

While these strategic disease intervention campaigns showcased remarkable successes compared to mass intervention approaches, they would not have been successful without the ability to rapidly detect and report the location of disease incidences and subsequently apply resources in a considered manner. Indeed, the routine reporting and geo-spatial mapping of health data using digital platforms are widely implemented throughout Africa, with some diseases (e.g., malaria and ebola) often having dedicated reporting channels within endemic countries [23,24,25,26]. Furthermore, the ability to monitor disease intervention activities so that the implementation of resources can be evaluated is equally important [24,27]. Based on these observations, one could thus argue that a strategic vaccination approach towards dog rabies elimination could be feasible if similar tools and resources were made available to resource-limited rabies-endemic countries. In the past, targeted vaccination campaigns have been used effectively during small-scale rabies outbreaks in endemic countries [3], but there remains limited evidence towards its feasibility on a larger scale.

The main aim of this manuscript was to provide clear evidence towards the feasibility of eliminating dog rabies through strategic interventions based on accurate and timely surveillance. To this end, we report on the development and implementation of tools and resources that have been specifically designed to facilitate the implementation of strategic dog vaccination events in resource-constrained rabies-endemic countries. In addition, we provide evidence showcasing the feasibility of this approach in the form of two distinct case studies, viz. Tanzania’s Unguja island and Zimbabwe’s metropolitan province of Harare.

## 2. The Rabies Epidemiological Bulletin and Its Role in Strategic Dog Vaccination Campaigns

The Rabies Epidemiological Bulletin (REB) is a free-to-use web-based data platform that was developed in 2016 by the Global Alliance for Rabies Control (GARC) in an effort the reduce the under- or inconsistent-reporting of rabies by endemic countries in Africa, the Middle East, and Asia [28,29,30]. The District Health Information System 2 (DHIS2) platform was chosen for the REB to align the envisaged rabies surveillance system with that of national health systems (often powered by the same platform) in more than 40 African countries [30,31,32]. The main purpose of the REB is to facilitate the establishment and routine use of One Health rabies-specific surveillance systems in endemic countries—providing governments with a system that would enable them to make data-driven decisions relating to rabies [30]. As such, by utilizing the system, national governments gain access to a free surveillance tool that is capable of generating various outputs including visuals, graphs, and tables that are updated with the latest data in near real-time. Most importantly, the data entered into the REB remains that of the national government and GARC (as the system developers) have no access to the data without express permission from the government authorities [30].

Since becoming operational in 2016, the benefits associated with the use of the REB has resulted in its use in more than 42 rabies-endemic countries around the world, with 17 of those countries benefitting routinely from the online platform and its functionality. While the REB was initially developed to collect, collate, analyse and disseminate national-level data—and still serves that purpose for any interested rabies-endemic country—it has also been continuously improved through the development of various add-on “components” that have been designed to collect sub-national data at any administrative level. These components allow users to improve the resolution of their surveillance data by providing information at a more localised-level while also automatically aggregating the data up the administrative hierarchy—easing data reporting and improving data quality at all administrative levels [33]. Two of these components—the Rabies Vaccination Tracker (RVT) and Rabies Case Surveillance (RCS)—have been developed to facilitate the implementation of strategic dog vaccination events and their use and applicability are discussed in more detail here.

### 2.1. Rabies Case Surveillance Component

The RCS component of the REB facilitates the development of interactive spatio-temporal maps that pinpoint the locations of both laboratory-diagnosed and suspect-rabies cases at a community-level by enabling the end-user to input the animal species, the diagnostic outcome, and the sample number for each diagnosed specimen. Once the metadata has been captured, each case is plotted by the system, in near real-time, on a map using either manually captured global positioning satellite (GPS) coordinates or an interactive map that can be used to identify the location within the community if no precise GPS coordinates are available. The RCS component enables end-users to upload surveillance data for any administrative level in the country after which the data is not only aggregated to the national level but also becomes usable data in the form of maps, graphs, and tables that enable decision-makers to identify rabies hotspots and high-risk areas where intervention campaigns can be implemented. The maps and graphs are all interactive, with users being able to select specific cases to reveal the exact information pertaining to that sample (i.e., the sample metadata is shown on the map for each sample), eliminating the need to find the sample in the database. In addition to the maps, the data entered using the RCS component also results in a cascade of notifications being sent to stakeholders informing them of the newly captured case so that action can be taken, ensuring timely responses to outbreaks and previously unidentified cycles. As eluded earlier, this surveillance information—and the subsequent identification of hotspots—was instrumental in driving successful intervention campaigns for the various diseases that have been either eradicated or successfully controlled.

### 2.2. Rabies Vaccination Tracker Component

Like the RCS component, the RVT component helps track mass vaccination data using maps and visuals as outputs. The RVT component enables end-users to either automatically import or manually enter the information for each vaccinated animal (e.g., species, age, sex, and GPS coordinates). Similar to the RCS component, an interactive map can also be used to obtain broad GPS coordinates if no grass-roots level data was captured with regards to the location of each animal. In addition to the maps, the data entered using the RVT component can also be used to populate graphs and other visuals that can be used to increase the government’s knowledge of its dog population and its ecology. System calculations are available to determine current vaccination coverage in terms of the estimated dog population for each area (based on the human to dog ratio), the sex and age ratios of vaccinated animals and a variety of other calculations important for actionable decisions.

In summary, both the RVT and RCS component serve to provide a valuable commodity to governments by overcoming the need for timely analysis and the costly GIS experts/software required to adequately analyse the collected data [34]. In addition to acting as independent elements on the REB, the two components can also be overlaid to create one interactive map showing rabies vaccination data in relation to the rabies-positive cases—a key element to the strategic allocation of valuable resources and targeted intervention.

## 3. The GARC Data Logger and Its Role in Strategic Dog Vaccination Campaigns

In most rabies-endemic countries, rabies vaccine distribution is monitored by comparing the number of doses of vaccine given to each vaccinator at the start of the day to the number of unused vaccines returned at the end of the day. While this approach provides a basic indication of vaccinated animals it does not provide an indication of where the vaccinated animals reside within the community nor does it allow stakeholders to effectively distribute limited resources to areas where it is needed most. To overcome these limitations and improve the resolution of the information collected during vaccination campaigns, methodologies aimed at collecting and monitoring grass-roots level data during vaccination events have recently become more prominent. Of these methodologies, mobile phone applications have been the most widely used and tested to-date [35,36,37]. While the DHIS2-powered REB is a primarily web-based instance, a mobile phone application is also integral to the system and the various add-on components, should the user choose to use that approach. However, relying on mobile phone technology is not without limitation [38,39] and thus we opted for an additional approach by developing a custom-made data collection device with the ability to record the most important data associated with every vaccinated animal in the simplest possible manner.

**Figure 1:**
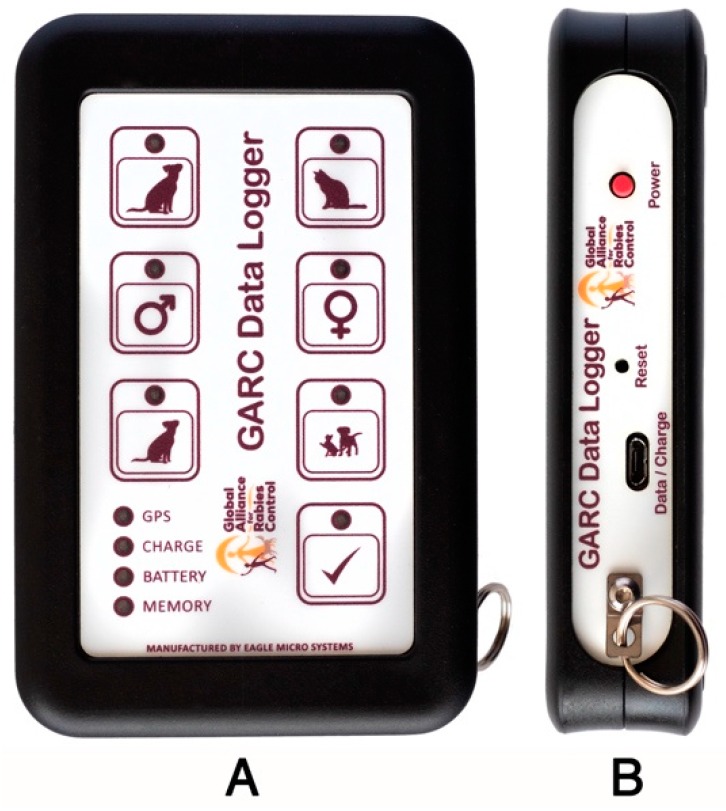
The front (**A**) and side (**B**) view of the Global Alliance for Rabies Control (GARC) Data Logger (GDL). Each picture on the front of the GDL device is a touch-activated button that has a corresponding LED that lights up when selected.

The GARC Data Logger (GDL) is a labile, hand-held data collection device that was custom-developed to facilitate the rapid collection and storage of dog vaccination data. To limit the wear and tear of the device, the GDL was developed using a hard-wearing plastic casing with touch-activated buttons and indicator light-emitting diodes (LEDs) (Figure 1). The lack of a liquid crystal display (LCD) screen not only means that the GDL device can be operated in bright sunlight without encountering glare, but also makes the device more resistant to damage should it accidentally be dropped by the user during routine use—in so doing keep the maintenance responsibility to a negligible level. Lastly, by relying on satellite triangulation to collect GPS coordinates, the GDL device provides high-resolution geo-spatial data anywhere in the world and is not reliant on mobile phone networks (through either cellular phone tower triangulation or data costs).

Being independent of mobile networks, the costs associated with the GDL only pertain to the initial purchase cost (comparable with affordable smartphones), with no additional running costs needed (except electricity for charging the device between use). In addition, as the GDL is sturdy and durable—evidenced by the fact that some devices have been operational for more than 16 months without breakages or a need for replacement—no additional costs relating to repairs or maintenance are required.

### 3.1. Capturing Data with the GARC Data Logger

When designing the GDL, it was not only the device’s external features that were designed to be as simple as possible but also the data capturing process. Indeed, for each vaccinated animal, the vaccinator would answer three basic questions pertaining to the animal by touching the corresponding picture/touch-activated button, viz. (1) dog or cat, (2) male or female, and (3) adult or juvenile (Figure 1A). Once all three questions have been answered, the data entry is confirmed by a fourth button (the “tick” button). Data confirmation, in turn, results in the GDL device automatically recording the exact latitude and longitude of the vaccinated animal while also capturing the precise date and time. The entire data collection and confirmation process takes a few seconds, after which all the recorded data is stored internally to ensure that the data remains localised until it can be downloaded by the end-user. While the amount of data that is collected by the GDL may seem limited (compared to the diverse dataset of information that could be collected using mobile phone technology), the information collected is the essential information required to track vaccination. Due to the collection of only essential information, using the GDL limits the time spent on data collection and frees up more time for vaccination—the end goal for any dog vaccination campaign. Additionally, as data collection is a simple and rapid process, the vaccinator themselves can collect the data, ensuring that costly human resources allocated to facilitate the data collection process (salaries, per diems, accommodation, etc.) can be allocated solely to vaccination.

### 3.2. Downloading Data from the GARC Data Logger

**Figure 2:**
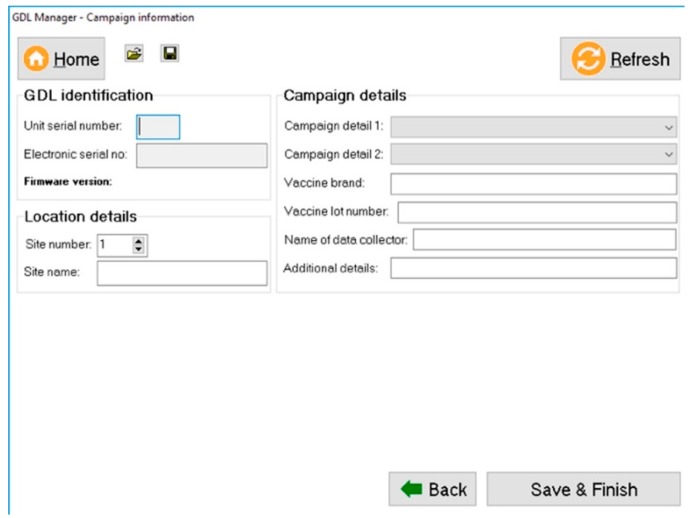
Screenshot of the “campaign details” screen of the GDL Manager software where data collected on the GDL can be supplemented with additional campaign information.

Once the vaccination work has been completed, the captured data is downloaded with a custom-developed software programme called the “GDL Manager”. The GDL Manager can not only download the collected data from the GDL that is plugged into any computer running on the Microsoft^TM^ Windows operating system but also enables the end-user to add additional campaign details to the collected data prior to downloading it. The supplementary data that can be added include the vaccine brand that was used, the vaccine’s lot number, and the specific vaccination site name, amongst others (Figure 2).

Downloading the data from the GDL results in the information being stored directly on the end user’s computer in a Microsoft^TM^ Excel and/or Comma-Separated Values (CSV) format. By first downloading the data to a computer, the end-user decides what happens to the data in terms of data sharing and distribution. Some end-users have access to skilled GIS professionals readily available and can thus have the data analysed and subjected to spatio-temporal plotting using independent software programs. Most often though, the stakeholders running dog vaccination campaigns do not have those resources readily available and would thus not be able to analyse their data in a timely manner. In such instances, the end-user can choose to add their data onto the REB where the GDL data is collated and analysed by the RVT component before maps and visuals are automatically generated by the system. Indeed, the data exported in the CSV format from the GDL is in the exact format required to ensure that the data can be automatically uploaded onto the REB, allowing the map and other visuals to be updated in near real-time without the need for skilled GIS experts or the additional costs associated with data analysts.

## 4. Case Studies of the Implementation of the GARC Data Logger and the Rabies Epidemiological Bulletin’s Novel Components

### 4.1. Implementing a Strategic Vaccination Campaign on Tanzania’s Unguja Island

The Zanzibar Archipelago, an extensive group of islands off of the eastern coast of Africa, is a semi-autonomous region of the United Republic of Tanzania, under the authority of the Revolutionary Government of Zanzibar. Based on anecdotal evidence, the Zanzibar Archipelago remained free from dog-mediated rabies until the 1990s, when the disease was detected on two of the biggest islands: Unguja island and Pemba island [40,41]. Of the two main islands, Pemba island was included in the Bill & Melinda Gates Foundation (BMGF) project where rabies was controlled, but not eliminated, by means of MDV campaigns between 2011 and 2014 [40,42,43]. At the same time, the Department of Livestock Development on Unguja island initiated a humane free-roaming dog control programme in collaboration with World Animal Protection (WAP). The “Rabies Control and Dog Management Project”, which relied on the implementation of MDV campaigns across the island between 2009 and 2015, resulted in the number of dog rabies cases declining, with what was suspected to be the last case of rabies being clinically diagnosed in 2015. Based on the decline in clinical rabies cases on the island, the government decided to embark on the rabies-free self-declaration process [44], which would have made Unguja island the first region in Africa to be declared free by means of dog vaccination. However, the apparent success of the “Rabies Control and Dog Management Project” could not be substantiated as Unguja island lacked diagnostic capacity, preventing them from subjecting any suspect rabies samples to routine laboratory diagnosis.

#### 4.1.1. Establishing Rabies Surveillance on Unguja Island

In 2016, GARC facilitated the establishment of a laboratory-based rabies surveillance system on Unguja island. To this end, animal health technicians were trained on the safe collection of brain stem and mixed brain samples [45], while local diagnosticians were trained on the implementation of the direct, rapid immunohistochemical test (DRIT) assay at the Veterinary Investigation Center (VIC) [46]. Following the establishment of the surveillance system, the REB was introduced to facilitate improved data collection, analysis and—with accurate laboratory diagnosis—further strengthen the surveillance system. As the REB is a freely available, web-based system, no specific or specialised equipment was needed and only basic training in its use was required. With the implementation of an effective rabies surveillance system, animal, and human rabies cases were detected across the island from 2016 onwards, with each case being captured on the RCS component on the REB by local campaign managers.

#### 4.1.2. Planning the Strategic Dog Vaccination Campaign on Unguja Island

In response to the detection of rabies cases across the island, GARC and the Department of Livestock Development started planning a vaccination programme that could be implemented with limited resources. In contrast to the “Rabies Control and Dog Management Project” that aimed to achieve sufficient vaccination coverage of the entire dog population (determined to be approximately 13,000 dogs by means of a survey undertaken in 2009), a strategic vaccination approach was considered. Through visually impactful outputs, the REB-generated spatio-temporal maps plotting rabies surveillance data-enabled hotspot areas to be identified. The impact of this methodology was two-fold: (1) It ensured that the vaccinators were directed to the high-risk areas; and (2) it resulted in the reduction of the amount of vaccine required as a 70% coverage was required in only the hotspot areas, equating to a smaller estimated dog population requiring vaccination (approximately 41% of the total estimated dog population).

#### 4.1.3. Implementing the Strategic Dog Vaccination on Unguja Island

**Figure 3:**
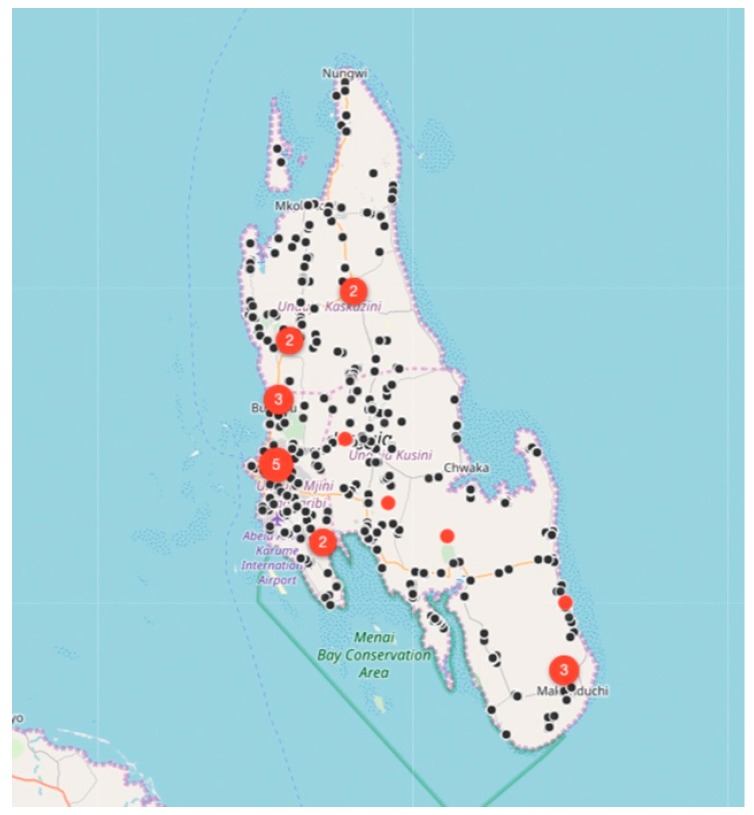
Maps of Unguja island showing the location of laboratory-confirmed animal rabies cases (red dots) and locations of vaccinated animals in 2017 (black dots). The number of rabies cases per location is displayed in terms of the size and the number within each red dot, while only the location of the vaccinated animals is displayed on the map (black dots) for ease of interpretation. On this specific map, the total number of black dots represent the 2900 animals vaccinated between August 2017 and December 2017.

**Figure 4:**
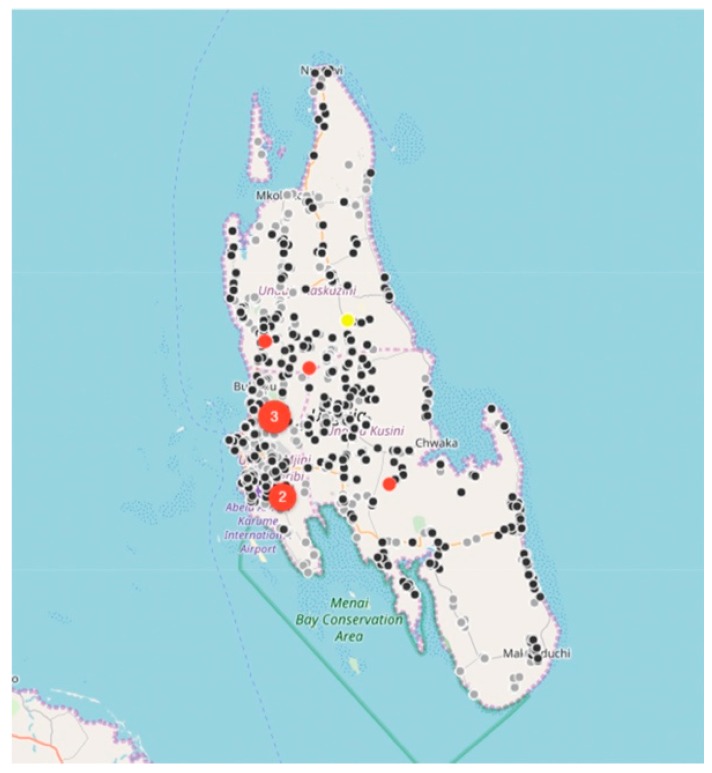
Maps of Unguja island showing the location of laboratory-confirmed animal rabies cases (red dots), clinically confirmed human rabies cases (yellow dots), locations of vaccinated animals in 2017 (grey dots) and locations of vaccinated animals in 2018 (black dots). The number of rabies cases per location is displayed in terms of the size and the number within each red dot. Only the location of the vaccinated animals is displayed on the map (grey and black dots) for ease of interpretation. On this specific map, the total number of grey and black dots represent the 6631 animals vaccinated between August 2017 and December 2018.

Between August 2017 and December 2018, the relatively small vaccination team consisting of 13 members, vaccinated 6174 dogs and 457 cats at selected fixed-point vaccination sites across the island with each vaccination being recorded using a GDL device (Figure 3 and Figure 4). While the number of dogs vaccinated over the course of the first 16 months of the program did not constitute a sufficient vaccination coverage according to the WHO recommendations (estimated vaccination coverage of 50% of the total population), the intervention strategy was successful in covering all of the priority areas as well as surrounding areas.

#### 4.1.4. Impact of the Strategic Dog Vaccination on Unguja Island

While rabies cases continued to be sporadically detected by the end of December 2018 (Figure 4), the number of rabies-positive cases in animals across the island had decreased by 71% (28 rabies cases in 2017 vs. 8 rabies cases in 2018). In addition to the observed decrease in rabies cases, we calculated the odds ratio for rabies positivity between the two periods (OR: 0.159) and used logistic regression to determine that there was an 84% (95% CI: 71–92%) lower likelihood of detecting a rabies-positive sample at the end of the strategic vaccination campaign in 2018. In addition to the statistical inferences presented here, the strategic vaccination campaign also impacted the epidemiological landscape of rabies across the island, with the remaining few cases largely being limited to the high-density urban settlements situated along the western coast of the island (Figure 4).

Based on the information provided here, the Department of Livestock Development of Unguja island is on track to control and eliminate dog-mediated rabies through the implementation of a second round of strategic dog vaccination in 2019/2020. If achieved, this would make Unguja island the first African region to comply with the OIE guidelines [44] that recommend the capacity and adequate disease surveillance required to undertake self-declaration of freedom from dog rabies and to further prevent the re-introduction of the disease onto the island.

### 4.2. Implementing a Strategic Vaccination Campaign in Zimbabwe’s Metropolitan Harare Province

Zimbabwe is a land-locked country in southern Africa that has been endemic for dog-mediated rabies since the 1950s [47,48,49]. Despite implementing annual dog vaccination campaigns across the country, an estimated 410 humans die of dog-mediated rabies in Zimbabwe every year [4,50]. While the majority of the animal and human rabies cases are limited to the rural areas of the country, the metropolitan capital city of Harare has been experiencing a persistent outbreak of dog-mediated rabies since 2010 [51]. In 2017, GARC—in collaboration with the Zimbabwean Department of Veterinary Services—worked towards gaining an improved understanding of the epidemiology of the rabies outbreak by using the RCS component of the REB to collect, collate, and analyse rabies surveillance data from the first eight years of the outbreak [51]. It was speculated that rabies had been introduced into the city on numerous occasions—with one introduction from the North-eastern region of the country resulting in its persistent transmission among the city’s dog population. Furthermore, it was found that approximately 80% of the rabies-positive dogs sampled within the city were considered owned animals of which the owners opted to forego annual rabies vaccination, most likely for financial reasons [51].

#### 4.2.1. Planning the Strategic Dog Vaccination Campaign in the Harare Province

Due to the limited resources and person-power preventing the vaccination of the city’s entire dog population (estimated to be approximately 230,000 dogs using the human to dog ratio (HDR) approach [52]), GARC and the Zimbabwean Department of Veterinary Services—in collaboration with the Zimbabwe National Society for the Prevention of Cruelty to Animals (ZNSPCA) and Veterinarians for Animal Welfare Zimbabwe (VAWZ)—planned and developed a strategic vaccination program relying on similar principles to those used in Unguja island. Using the RCS component of the REB to analyse outbreak data collected from 2010 onwards, several densely populated suburbs in the city were identified as high-risk areas maintaining rabies transmission. While those areas were prioritised for strategic vaccination, any other rabies cases reported to the REB were flagged for immediate ring vaccination to prevent further spread. In addition to responding to specific outbreaks within the city, the program also focused on a more sustainable and proactive response through the development of vaccination barriers in the peri-urban settlements to the North-East of the city to prevent further disease re-introductions.

#### 4.2.2. Implementing the Strategic Dog Vaccination in the Harare Province

**Figure 5:**
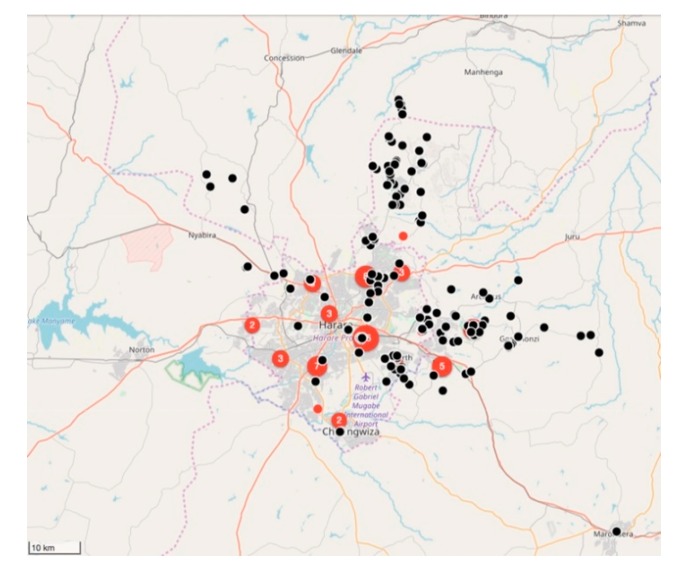
Map of Harare showing the location of laboratory-confirmed rabies cases (red dots) and locations of vaccinated animals (black dots) in 2018. The number of rabies cases per location is displayed in terms of the size and the number within each red dot. Only the location of the vaccinated animals is displayed on the map (black dots) for ease of interpretation. On this specific map, the total number of black dots represent the 9617 animals vaccinated between February 2018 and December 2018.

**Figure 6:**
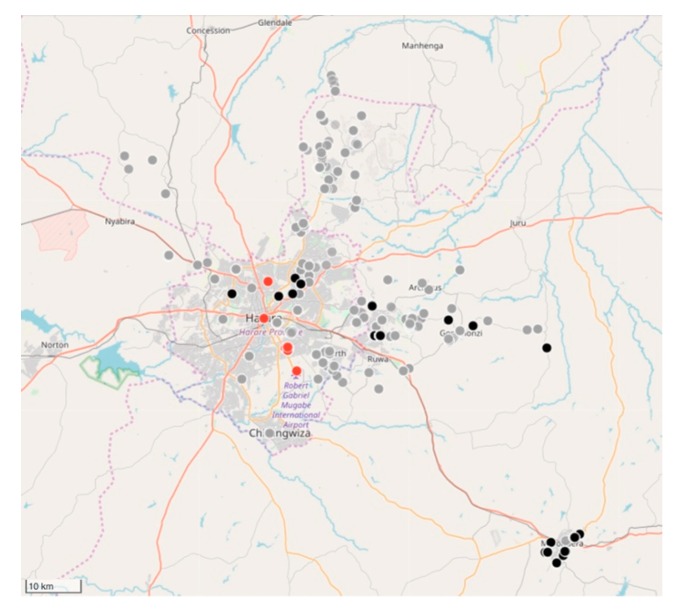
Map of Harare showing the location of laboratory-confirmed rabies cases (red dots), location of vaccinated animals in 2018 (grey dots) and the locations of vaccinated animals in 2019 (black dots). The number of rabies cases per location is displayed in terms of the size and the number within each red dot. Only the location of the vaccinated animals is displayed on the map (grey and black dots) for ease of interpretation. On this specific map, the total number of grey and black dots represent the 11,784 animals vaccinated between February 2018 and March 2019.

Between February 2018 and March 2019, the relatively small vaccination team consisting of 10 members, vaccinated 11,699 dogs and 85 cats (Figure 5 and Figure 6). Of the total number of vaccinated animals, 3135 (27%) were vaccinated within the metropolitan city (strategic intervention), while 8564 (74%) were vaccinated in the peri-urban settlements surrounding the city (vaccination barriers).

#### 4.2.3. Impact of the Strategic Dog Vaccination the Harare Province

Despite only achieving an estimated 5% vaccination coverage of the city’s entire dog population, a marked drop in the percentage rabies-positive samples had been observed by the end of 2018 (from 46% in 2017 to 33% in 2018). In addition to the reduced rabies positivity, we calculated the odds ratio for rabies positivity between the two periods (OR: 0.637) and used logistic regression to determine that there was a 36% (95% CI: 21–48%) lower likelihood of detecting a rabies-positive sample at the end of the vaccination campaign in 2018. Furthermore, we speculate that the benefit of the strategic vaccination campaign could have been far greater had it not been due to political disturbances [53] and disease outbreaks [54] that had occurred through the year in the target area. The prevailing conditions in the city continuously prevented vaccinators from responding to outbreaks, in turn, preventing at-risk dog populations from being vaccinated. Irrespective of these challenges, the project highlighted the efficacy and merit associated with a targeted, strategic vaccination campaign in a resource-limited setting. The success of the strategic rabies vaccination work in Zimbabwe is made even more evident when considering that the work was carried out by a small workforce that was not solely focussed on rabies control, but also had other animal welfare issues and disease outbreaks to tend to. This formula is aligned with those of other low- and middle-income countries where human and financial resources are typically limited, resulting in personnel being responsible for a multitude of diseases with little time to be allocated to a single disease such as rabies.

## 5. Conclusions

With the target date for eliminating dog-mediated human rabies by 2030 fast approaching [55,56], time is running out for rabies-endemic countries to act if they wish to successfully reach the goal before the deadline. Despite the identification of rabies as a priority disease in many African countries [57,58], a distinct lack of successful rabies elimination programmes from here suggests that African countries are not getting the government funding and support required to implement MDV campaigns of the scale and intensity needed to reach an adequate proportion of the total dog population [15]. As a result, dog-mediated rabies continues to be a scourge on the African continent and alternative strategies towards rabies elimination need to be considered. Lessons learned from other diseases that have either been eliminated or successfully controlled, include the validation of strategic interventions (vaccination) as a viable approach to rabies elimination in resource-limited countries.

As reported in this manuscript, the ability to implement strategic dog vaccination is firmly rooted in the ability to report rabies cases from the community-level with these data not only being geographically defined but also being disseminated to the responsible authorities so that disease intervention campaigns can be initiated in a timely manner. While the use of independent software programmes and manual notification channels could, in theory, provide governments with this much-needed information, the freely available RCS component of the REB has been demonstrated to provide all of the functions in near real-time while not requiring capital investments, extensive training or dedicated subject matter experts. These benefits were demonstrated in the two different case studies described here, each demonstrating how the RCS component of the REB was used to identify suspected or confirmed rabid animals before notifying the responsible authorities that the area should be earmarked for strategic vaccination. In addition to geographically defining suspect or confirmed rabid animals, strategic dog vaccination also relies on the ability to track each vaccination event (i.e., location of the vaccinated animal) by also recording metadata that is beneficial to understanding the ecology of the dog population in question.

While various methodologies have been developed to collect information during vaccination events using either paper-based forms or mobile phone applications, we specifically demonstrated the use of the custom-developed GDL, while also demonstrating how the interoperability with the freely available RVT component of the REB enabled the data to be uploaded and visualised in near real-time. This approach not only ensured that the governments were in control of their own data, but also allowed them to visualise the work done by the individual vaccinators, direct vaccinators to additional areas that were still at-risk, and closely monitor vaccine usage. In this way, the rabies programmes could be strategically overseen and controlled by a relatively small working group of professionals.

While the methodology and findings presented in this manuscript have shown the value of strategic dog vaccination in resource-limited settings, all of these areas would need to persist with this approach until elimination is achieved. The significant successes demonstrated through local-area campaigns should provide strong grounds for continued government support and commitment to rabies elimination. Such commitment will be essential for any nation to achieve zero dog-mediated human rabies cases by 2030.
